# Welcome to volume 3 of *Future Science OA*

**DOI:** 10.4155/fsoa-2016-0075

**Published:** 2017-01-05

**Authors:** Francesca Lake

**Keywords:** biomedicine, health, medicine, research

## Abstract

Happy New Year to all of our readers! Welcome to volume 3 of *Future Science OA*. 2016 was another excellent year, with us receiving some superb content for publication, and our becoming indexed on both Chemical Abstracts and the Emerging Sources Citation Index, meaning our content now appears on Web of Science. We thank our readers, reviewers, authors and Editorial Board members for their continued support, and look forward to working with everyone in 2017.

**Figure 1.**
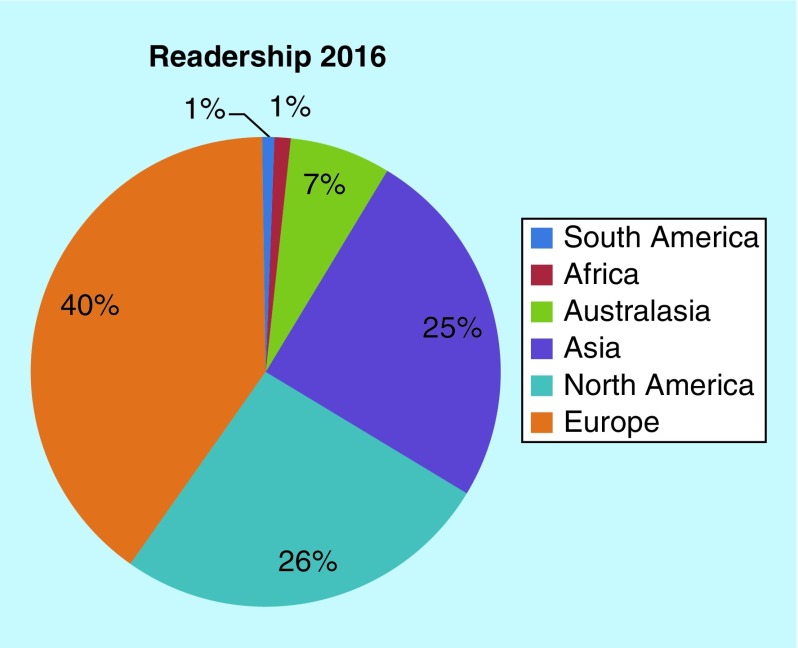
***Future Science OA* reader demographics 2016.**

**Figure 2.**
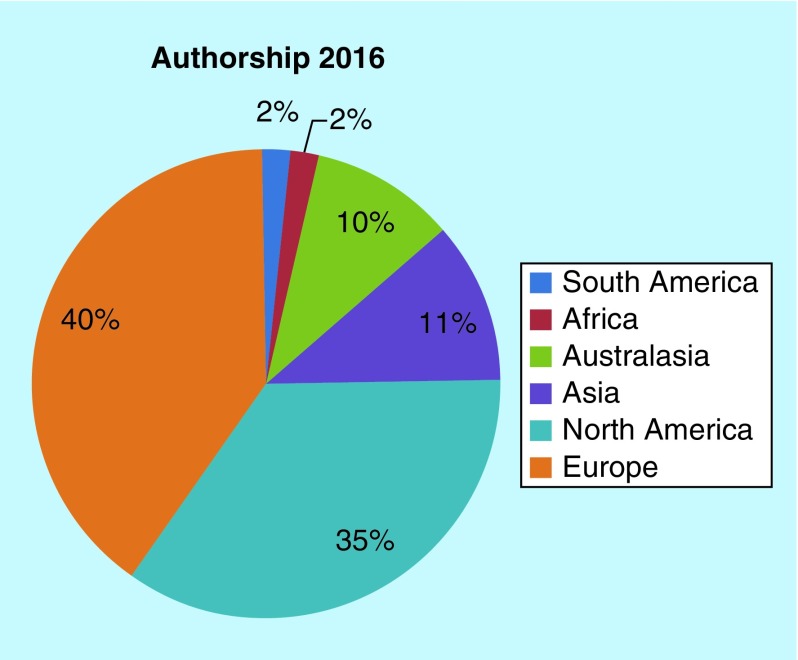
***Future Science OA* author demographics 2016.**

## Content highlights of 2016

Our most-read article of 2016 at the time of writing (October 2016) was an opinion piece from John Read and colleagues from the University of East London (UK) and the Hearing Voices Network [1]. In this article, the authors discuss the concept that the psychiatry profession is in crisis, and would benefit from re-evaluation. This article proved controversial and led to some interesting discussion [2].

Alzheimer’s disease research also featured prominently, with *Future Science OA* Editorial Board member, Pablo Moscato and his colleagues from the Hunter Medical Research Institute (NSW, Australia) proposing a new multivariate approach to cognitive classification [3]. The review from Ruth MacLeod and colleagues (University of Glasgow, UK) on the secretases in Alzheimer’s disease from Volume 1 also maintains its position in our most-read list [4].

Moving to the field of biomaterials, William G Whitford (GE Healthcare Life Sciences, UT, USA) and James B Hoying (Advanced Solutions Life Sciences, KY, USA) provided definitions for the novel terms used in bioprinting, in a bid to standardize the field [5].

2016 also saw us publish interviews with six of our early career researcher panel [6]. Heloisa Helena Milioli, Amy Dawson, Eleftheria Anastasopoulou and Ruth Bower discussed the challenges they have faced while pursuing their PhDs. Cath Martel and Kavita Beri, both making strides in their careers, discuss the lessons they have learned and provide their career advice for other aspiring researchers.

We continue to work with Altmetrics, and special mention goes to the following articles:

‘Physical activity and sarcopenic obesity: definition, assessment, prevalence and mechanism’ (Lee *et al.*) [7] – Altmetric score 99;
‘The effect of frailty should be considered in the management plan of older people with Type 2 diabetes’ (Abdelhafiz, Koay and Sinclair) [8] – Altmetric score 62;
‘Folic acid supplementation: what is new? Fetal, obstetric, long-term benefits and risks’ (Moussa *et al.*) – [9] Altmetric score 51;
‘Risk of hospitalization in patients with diabetes mellitus who have solid-organ malignancy’ (Karlin *et al.*) [10] Altmetric score 46.

## Demographics of contributors

With the journal read over 3500-times a month on average, 2016 has also seen an increased fraction of Australasian, Asian, African and South American readers compared with 2015 [11], which is excellent (Figure 1). While our authors remain spread similarly across the globe, we have seen the introduction of authors from Africa, which is tremendous to see (Figure 2).

## Editorial Board

We have seen an expansion of the Editorial Board this year, with the additions of Jürgen Bajorath (University of Bonn, Germany) and Jae-Young Lee (GIST, South Korea). What is more, we were delighted to promote XiuJun Li (UTEP, USA), formally of our Young Ambassador panel, to the full Editorial Board.

## Article outreach

We continue to post about our articles on Twitter (@fsgfso), Linkedin [12] and Facebook [13]. However, higher readership is consistently seen by our articles that have been shared by the authors. We have therefore created a handy guide to authors looking to improve their dissemination skills [14]. We are also pleased to continue our partnership with Kudos [15] in 2017, to support our authors in sharing their work and tracking the results of their activities.
